# Mapping the “catscape” formed by a population of pet cats with outdoor access

**DOI:** 10.1038/s41598-022-09694-9

**Published:** 2022-04-08

**Authors:** Richard Bischof, Nina Rosita Hansen, Øyvind Skarsgard Nyheim, Astrid Kisen, Lillian Prestmoen, Torbjørn Haugaasen

**Affiliations:** Faculty of Environmental Sciences and Natural Resource Management, Norwegian University of Life Sciences, Høgskoleveien 12, 1432 Ås, Norway

**Keywords:** Ecology, Conservation biology, Urban ecology

## Abstract

The domestic cat (*Felis catus*) is among the most popular companion animals and most abundant carnivores globally. It is also a pet with an immense ecological footprint because even non-feral and food-subsidized cats can be prolific predators. Whereas knowledge about the spatial behavior of individual domestic cats is growing, we still know little about how a local population of free-ranging pet cats occupies the landscape. Using a citizen science approach, we GPS-tagged 92 pet cats with outdoor access living in a residential area in southern Norway. The resulting position data allowed us to construct both individual home range kernels and a population-level utilization distribution. Our results reveal a dense predatory blanket that outdoor cats drape over and beyond the urban landscape. It is this population-level intensity surface—the “catscape”—that potential prey have to navigate. There were few gaps in the catscape within our residential study area and therefore few terrestrial refuges from potential cat predation. However, cats spent on average 79% of their outdoor time within 50 m to their owner’s home, which suggests that the primary impact is local and most acute for wildlife in the vicinity to homes with cats. We discuss the catscape as a conceptual and quantitative tool for better understanding and mitigating the environmental impact of domestic cats.

## Introduction

Pet cats with outdoor access are as much part of ecological communities as wildlife species that tolerate or benefit from proximity to humans. Despite receiving food subsidies from their human owners, many domestic cats are prolific predators and prey on a wide range of wildlife species^1,2^. With an estimated 600 million pet cats worldwide^3^, this is one of the most abundant carnivore species, wild or domestic. Consequently, the inferred quantity and the diversity of wild prey killed by domestic cats is staggering^1,2,4^. With the caveat that the reliability of extrapolated national or landscape-level numbers of cat-killed vertebrates can be drawn into question^5^, estimates range into the millions^6^ and even billions^4,7^ of birds and mammals annually.

The direct ecological impact of pet cats is linked with their outdoor activity, and a substantial proportion of pet cats with outdoor access hunt (e.g., between 50 and 80% in a study by Loss et al. in the USA^4^). A growing number of studies— often using GPS tracking—seeks to elucidate cat space-use behavior. Studies range from local^8^ to regional^9^, including an extensive investigation involving over 900 individual cats from 6 countries^3^. Thanks to such studies, knowledge about the space-use patterns of outdoor cats is growing. One consistent pattern revealed by telemetry studies of domestic cats is that of substantial regional and individual variation in spatial behavior, such as home range size, the propensity to roam, and habitat selection. This variation can, at least in part, be attributed to differences in intrinsic characteristics of the cat-and-owner entity (e.g., cat demography and maintenance) and the outdoor environment (e.g., habitat). For example, age, sex, and sterilization status influence home range size, as does the position of the cat’s home along the urban-nature gradient: younger, male, intact cats, and cats in rural areas tend to have the largest home ranges^3,10,11^.

Individual organisms and their impacts scale up to populations. The same is not always true for ecological studies; information based on a small subset of individuals (or individuals originating from a patchwork of sites) are notoriously unreliable for scaling up to population-level inferences^12,13^. Regardless of the reasons behind individual and spatial heterogeneity, sparse or non-representative sampling prevents trustworthy inferences about population and landscape-level cat space-use and predation on wildlife. We are not aware of any study that has attempted to track all or the majority of cats within a neighborhood with a typical cat density. This is a surprising gap in information, as the local ecological impact of pet cats is attributable to their sheer numbers^14^.

**Figure 1:**
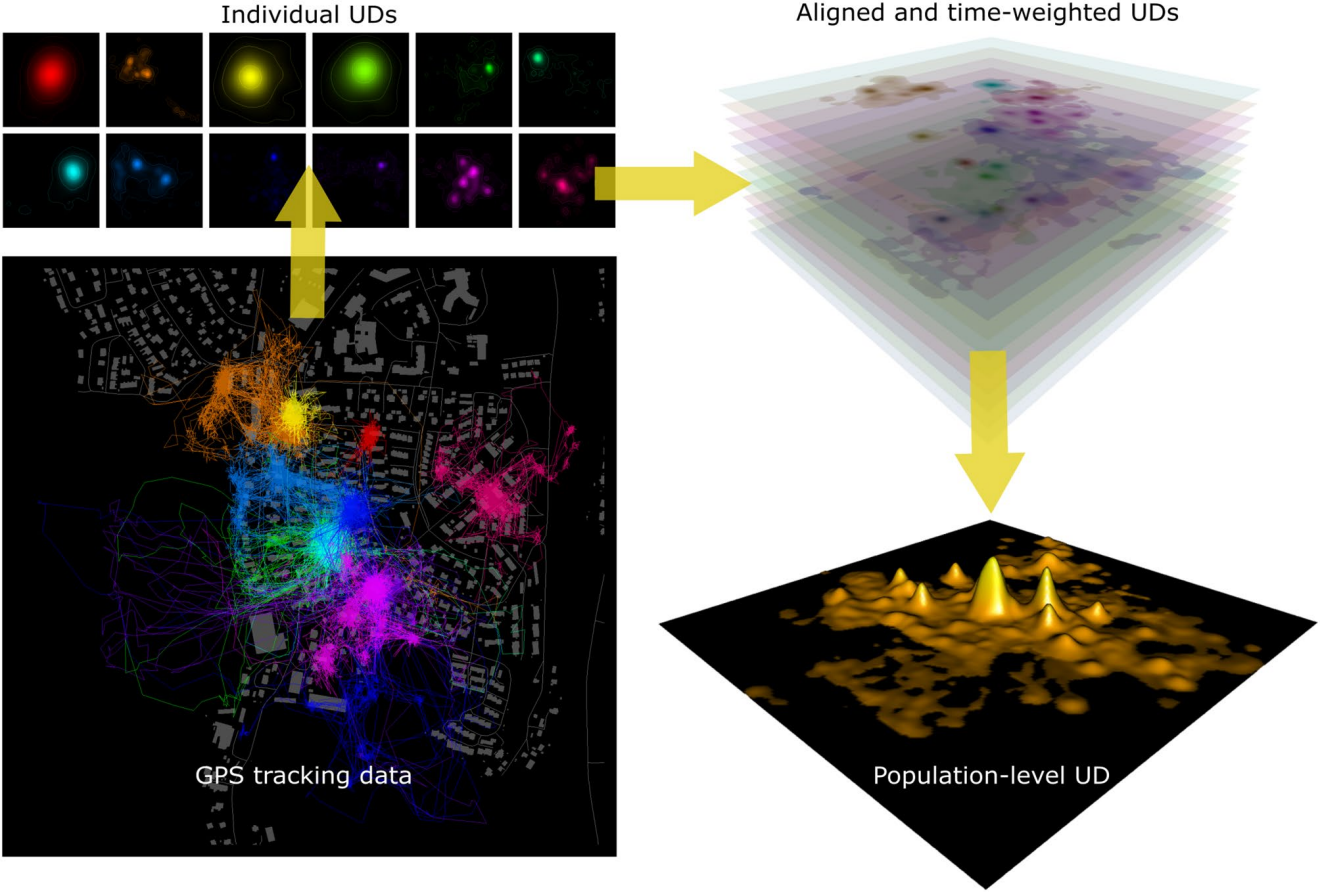
Illustration of the construction of the catscape by aggregating the utilization distributions (UDs) of 12 example pet cats in a suburban neighborhood (buildings shown in grey). High-throughput GPS data (**A**) are used to estimate individual UDs with Brownian bridge motion models (**B**). Individual UDs are weighted according to the average proportion of time spent outdoors on days with data (**C**) and are then summed across individuals to yield the combined intensity of use (**D**). All maps (2D and 3D) were created using R^17^.

Wildlife preyed upon by cats face the combined local cat population, which in urban areas readily reaches densities of several hundred individuals per km^2^^5,15,16^. Mapping the “catscape”—the combined intensity of space-use by a cat population—could help us better understand and potentially mitigate the ecological impacts of domestic cats. During a month-long study, and with the help of citizen scientists, we GPS-tagged and tracked 92 domestic cats living with their owners in a small (1.1 km^2^) residential area in southern Norway. This unprecedented population coverage allowed us to construct a population-level utilization distributions (Fig. 1), an ecologically meaningful representation of outdoor cat populations: the spatially-explicit risk of encountering cats across the landscape. The catscape, if not equivalent to the risk of predation, is likely linked to it.

## Results

### Study cats

The 92 cats included in this analysis were associated with 76 households; 14 households had 2 participant cats and one household had 3 participant cats. The sex ratio of GPS-tagged cats was close to even (49% female), with similar median ages for the sexes (females: 5.5y, males: 5y). All except 2 juvenile cats (1 female, 1 male) were reportedly sterilized. Thirty-two percent of cats had free or partially free outdoor access via a pet door/flap; the remaining individuals were released manually by their owners. The majority of cats (78%) were given unlimited access to food. Owners did not provide demographic and maintenance details for 6 cats; percentages provided above refer to cats for which information was provided.

We estimate that we tracked $\ge 73\%$ of the cats (126) inferred to be living inside the study area and approximately 44% of the outdoor activity by all cats. These estimates are based on initial registrations by cat owners to the project (some of which chose not to participate), information from participating cat owners about other cats they knew in their neighborhood, and ancillary information from 47 camera traps distributed throughout residential backyards in the study area (Extended Data Fig. S1). Three cats that were recruited within the study area but did not provide 5 days of GPS tracking data were excluded from the analysis. Activity missed is likely a combination of activity by participant cats during times when they were not wearing a functioning collar, activity by non-participant cats that live inside the study area, and activity by cats entering the study area from outside. To our knowledge, all cats using our study area are owned cats.

### Movements and home ranges

**Figure 2:**
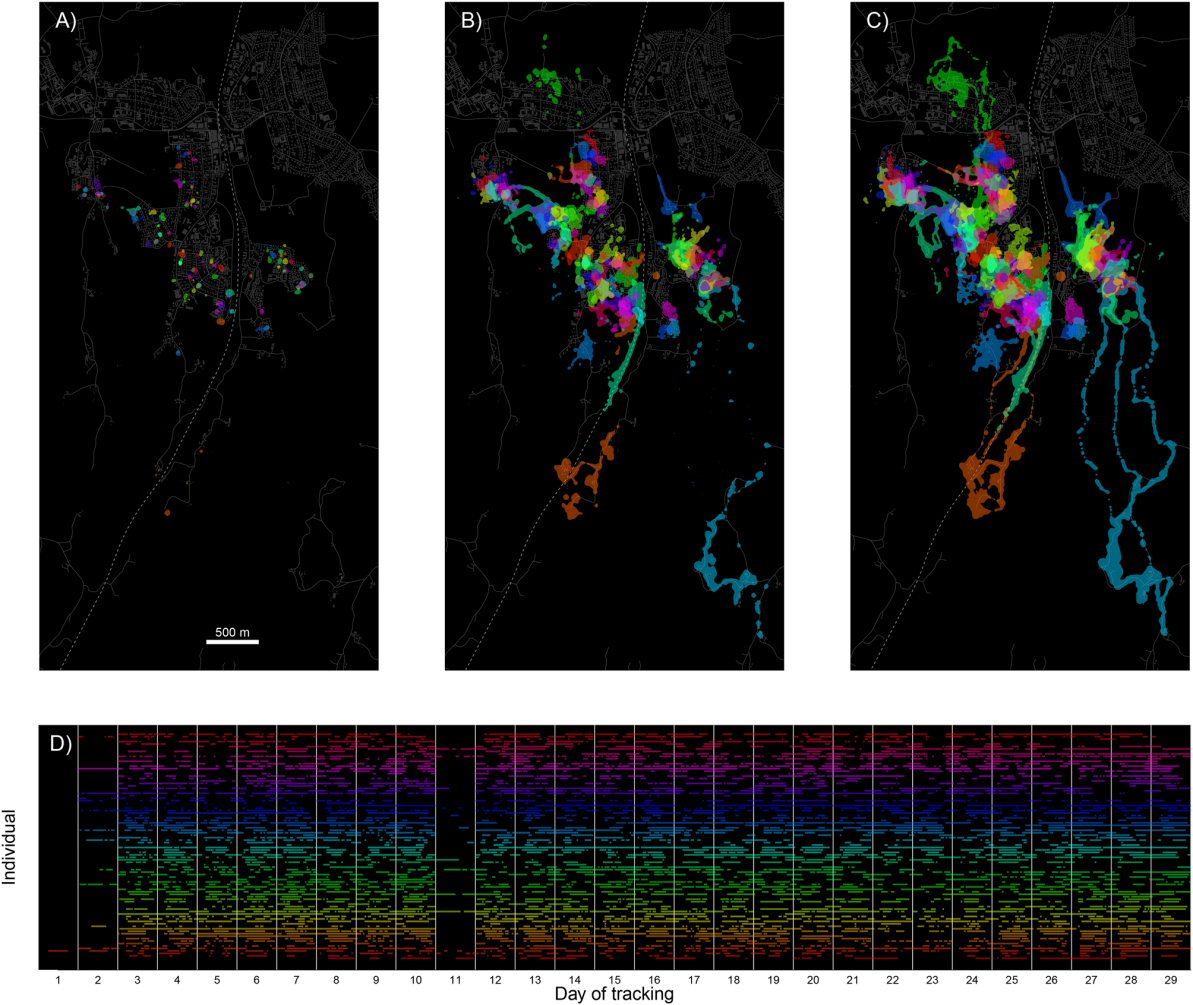
Individual home ranges and temporal tracking coverage for 92 pet cats (color-coded) that were GPS-tracked during May 2021 in a residential area in southern Norway. Shown are 50% (**A**), 95% (**B**), and 99% (**C**) home ranges, based on corresponding vertices of Brownian bridge movement models. Cats spent the majority of their outdoor time in close proximity to their owner’s home, evident in the small core areas (50% home range) compared with the 95% and 99% home range polygons. Grey lines and polygons in the background indicate roads and buildings, respectively. A fenced railroad (dashed line) transects the study area. Panel (**D**) shows the temporal coverage of outdoor GPS tracking data of each cat (rows). A pronounced gap in GPS tracking on day 11 coincides with temporary retrieval of GPS units to download initial data and check for technical problems before continuing tracking. The figure was created using R^17^.

We included on average 21.4 days (range: 6–28; interquartile range [IQR]: 19–26) with GPS position data from cats in the study. The average daily outdoor tracking duration was 8.4 h (range: 1–18.2; IQR: 5.6–11.2; Fig. 2D). Home range size estimates (95% BBMM) during the study period ranged from 0.3 to 22.1 ha (mean: 2.6; IQR: 0.7–3.2; Fig. 2B). Female home ranges averaged 1.5 ha (range: 0.3–5.4; IQR: 0.5–2), compared with 3.7 ha (range: 0.6–22.1; IQR: 1.2–3.8) for males. At 0.2 ha (range: 0.1–0.8; IQR: 0.1–0.2; Fig. 2A), the core area (50% UD; Fig. 2A) of the average cat was 1/14 the size of its 95% home range, Fig. 2B). Individual cats spent on average 79% (32–100 %) of their time outdoors within 50 m of their owner’s home. The overall average distance of outdoor positions from the home was 58 m (range: 6–914; IQR: 13–48), the average maximum distance was 352 m (range: 48–3384; IQR: 134–401). The homes of cats in this study were on average 76 m (range: 1–245; IQR: 19–115; SI Fig. S1) from the urban edge, and home ranges (95% BBMM) included on average 19% non-urban areas (range: 0–77; IQR: 0–73).

### Population-level UD

**Figure 3:**
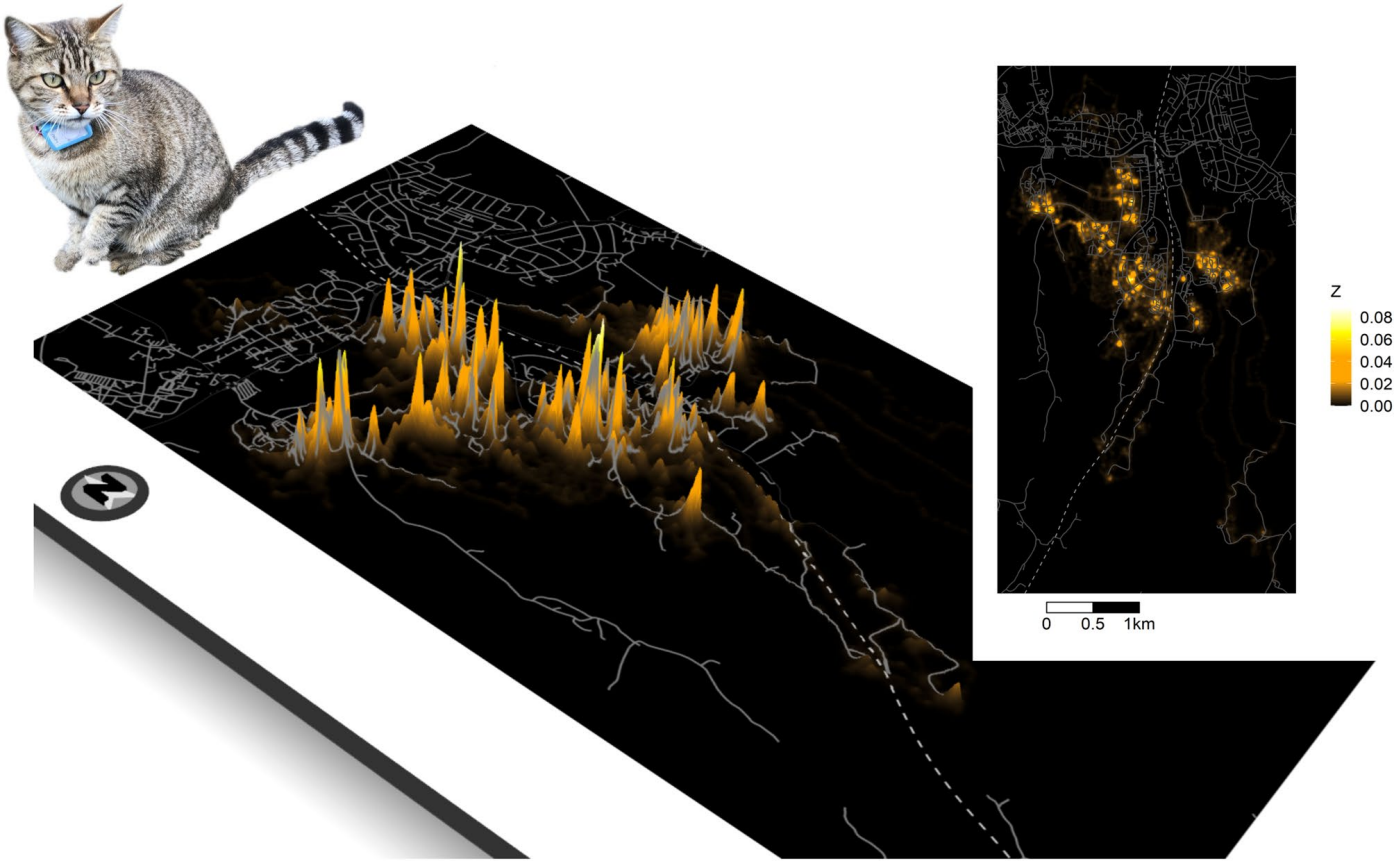
Three-dimensional representation of the catscape (with 2D inset), constructed using individual utilization distributions of 92 domestic cats. The height of the surface (*Z*, square root transformed for visualization) denotes the intensity of use by cats. Grey lines indicate roads; a fenced railroad (dashed line) transects the study area. Both maps were created using R^17^; cat photo by R. Bischof.

The surface representing the population-level UD was highly variable; within the region delineated by the 99% vertex of the catscape, we measured a more than 1800-fold difference between the highest and lowest utilization intensity values (Fig. 3). At the population-level, cat utilization intensity was 11-fold higher within 50 m of a cat owner’s home than at a distance of 50 m to 100 m. Thirty-seven percent (0.59 km^2^) of the area covered by the catscape (1.59 km^2^ based on the area delineated by the 99% volume contour) extended beyond urban and other developed areas (e.g, farm yards, etc.). However, only 9% of the utilization intensity associated with the catscape fell outside urban and developed areas.

## Discussion

Our analysis revealed that individual utilization distributions (UDs, Fig. 2) of pet cats with outdoor access coalesce into a joint surface that drapes over the suburban landscape and the surrounding areas (Fig. 3). The height of the surface is a function of both the number of cats that use a particular location and the amount of time they spend there. It is this surface that potential prey species have to navigate, and it not only conveys the spatial configuration of the risk of encountering a pet cat, but its characteristics can also guide the selection and targeted application of mitigation measures.

The population-level UD reflects spatial variation in relative utilization and potential impacts of domestic cats within a study area. It can also be used to compare different areas and populations, assuming similar coverage (proportion of the population and activity tracked). Although we tracked the majority of cats living within the study area, the catscape shown here represents a minimum use-scenario, as not all outdoor activity by cats was tracked. Nonetheless, spatio-temporal coverage for a cat population of such density was unprecedented (Fig. 2D).

Although seemingly high, the inferred number of cats (126) with homes in our study area (1.1 km^2^) is comparable or lower than values reported elsewhere. For example, domestic cat densities in urban areas in the UK have been estimated to range between 131.8 and 1579.2 cats/km^2^ (median 417.3 cats/km^2^)^15^ and average cat densities of above 200 per km^2^ have been reported by studies in New Zealand^5^ and the US^16^. In Norway, with a human population size of 5.4 million, there are an estimated 770,000 pet cats^18^.

One approach to mitigate environmental impacts of cats is to decrease the spatial coverage and overall height of the catscape. This could be accomplished by reducing the number of cats with outdoor access, including prohibiting cat ownership in sensitive areas^19^, or limiting the amount of time cats spend outdoors^20^. Reduction in pet cat numbers, while theoretically feasible, presumably involves the kind of political and regulatory maneuvering most jurisdictions shy away from^21^. Moreover, without a better understanding of the role of intra-species interactions in the spatio-temporal dynamics of cat populations, it is unclear whether the reduction of cat numbers or the duration and frequency of outdoor access would lead to a corresponding reduction in overall space utilization^19^. It has been suggested that removing cats in a dense population may lead to a reduction in predation pressure without a reduction of spatial coverage or creation of cat-free areas, as remaining cats expand their home ranges due to density-dependent effects on movements^5^. Counter-intuitive effects of predator control on predation have been demonstrated or suggested in other species. For example, predator control can increase predation through the disruption of social structure when dominant individuals are removed, due to changes in the behavior of remaining individuals, or through knock-on effects on other predator species that are released from competition^22^.

The spatial coverage of the catscape could also be reduced by shrinking the average home range size of constituent cats. Confinement^20^, including curfews to keep cats indoors during times with more pronounced roaming behavior^8^, and sterilization^11,23^ have been suggested to reduce roaming behavior and home range size in domestic cats. This, like a reduction in the number of cats, could decrease the total size of the area impacted and create gaps in the catscape to serve as refuges for potential prey. Note that all but two cats in our study were sterilized, so the catscape shown here already exemplifies a best-case scenario in terms of reproductive control effects on range of movement.

The catscape is characterized by pronounced peaks (Fig. 3), reflecting high site fidelity of cats to their owners’ homes, with further clustering evident in both individual and population-level UDs (Fig. 3). Cats in our study spent 79% of their time outdoors within 50 m of their owner’s home, and concentrated activity and small home ranges evidently characterize the spatial behavior of most pet cats^3^. This suggests that the highest predation risk—or at least the highest risk of encountering a cat—is local and most acute for wildlife utilizing space in the vicinity of residences with cats. The configuration of the catscape into pronounced hot-spots offers opportunities for more targeted mitigation measures. This could involve the removal or enhanced protection of bird feeders, nest boxes, and bird baths near the homes and in the yards of cat owners. Here too, unintended consequences are possible if, for example, the reduction in predation opportunities leads to an expansion of the cat’s activity area, with risk simply shifting farther away from the cat’s home. To our knowledge, all cats using our study area are owned cats. Feral cats tend to have larger home ranges and predation impact than owned cats^4,24^, and the presence of feral cats in other locations likely changes the spatial configuration of the population-level UD, as well as the associated environmental impacts.

### Beyond urban

Thirty-seven percent of the catscape extended beyond urban areas and areas with infrastructure (roads, farm yards, etc.). That domestic cats living in urban areas range into natural areas has already been demonstrated^3,25^. Our study revealed the magnitude of this halo at the population-level and the highly variable contribution that individuals make to it, with 10% of participant cats accounting for 62% of the non-urban habitat use. However, because cats in this study lived primarily in an urban setting and individual space utilization declines rapidly from each cat’s core use area (Fig. 2), the majority (91%) of the risk of encountering a cat fell into urban habitat. The relationship between the population-level UD and predation risk to wildlife is likely modulated by habitat and its configuration within the home range of individual cats. For example, individual cats living near and using non-urban areas have been reported to have different movement behavior and be more prone to prey on certain wildlife^26,27^. It is thus possible that predation risk for some wildlife species is greater in the peripheral, lower-amplitude regions of the population-level UD, if these represent roaming areas that extend into non-urban habitat. However, like wildlife studies on predation^28^, studies on predation by cats tend to focus on kill rates—or apparent kill rates—rather than predation rates and thus quantify predation from the perspective of the predator (number of prey killed or returned by a cat per unit time) instead of that of the prey (risk of being killed or proportion of the prey population taken). It is unclear to what extent apparent habitat-kill/return rate relationships translate into spatially-explicit risk for prey (predation rate).

Methods that decrease cat density and home range size/ranging behavior would potentially also reduce the extent to which the catscape and associated impacts bleed into non-urban areas. Another, likely unpopular, strategy could be to tolerate medium and large carnivores around urban areas, as these may provide an added ecological service through culling of roaming and stray cats or impacting cat space-use behavior^29–32^ by creating a landscape of fear^33,34^. Regarding the latter, it is worth noting that Kays et al.^3^ found no evidence of movement restriction of owned cats in response to the presence of a larger predator (coyotes, *Canis latrans*).

### Individual variation

Individual variation in space-use was high among cats in this localized population. This included variation in home range sizes, the extent to which home ranges included non-urban areas, and the degree of spatial association with the owner’s home. High intraspecific variation in cat space-use behavior has been reported by others^3,10^ and likely propagates to variation in environmental impacts. Substantial individual variation in space-use and other behaviors poses a critical obstacle for the common approach in wildlife ecology of scaling inferences to the population-level from a limited sample of individuals. To be representative, the sample would have to capture the population-level breadth and distribution of the parameter of interest, which is difficult to achieve and verify^35^. Furthermore, investigations targeting individual interactions or the manifestations thereof (e.g., territory configuration and turn-over) are impeded when the majority of individuals remains hidden from the observer; the behavior of tagged individuals is interpreted with the sample in mind, whereas in reality it is influenced by additional, unobserved animals.

Due to individual variation in space-use, mitigation efforts may be more effective if they are customized based on individual characteristics. Inexpensive and accessible pet tracking devices now make it possible for most owners to remotely monitor the outdoor movements of their pets. This offers opportunities for cat-specific mitigation measures that owners can implement based on information about their cat’s movements and in relation to specific husbandry practices. At a minimum, owners could employ selective containment of cats that exhibit roaming behavior. Owners could also experiment with and adopt maintenance regimes (diet, behavioral enrichment^36^) that reduce roaming behavior of individual cats, even if the impact of these as general interventions may be questionable^10^.

### Scaling-up monitoring through citizen science

In our study, the synchronous collection of spatial data on a large proportion of cats in one area was achieved through the participation of the owners of these cats, that is, citizen scientists. Data collected at or near the population scale can help answer important questions that remain elusive to studies with small samples or samples scattered over vast areas. Relevant topics concerning domestic cats include the identification of determinants of space-use and movement that take into account intraspecific interactions and, eventually, measuring the link between space-use and predation pressure exerted. A particularly important line of inquiry concerns the systematic assessment of the impact of mitigation measures at the population scale.

Citizen science can be an efficient and cost effective approach for collecting data at scales that are otherwise difficult to attain. The associated limitations in control and inherent biases may to some extent be estimated and accounted for by accompanying independent survey methods, such as camera trapping in our study. Citizen science can also serve as a vehicle for deciding, motivating, informing, and evaluating measures for mitigating the ecological impacts of cats, as most interventions will need to be implemented by the cat owners themselves. We agree with Crowley et al.^21^ that, given the multitude of circumstances faced and attitudes exhibited by cat owners, multidimensional and customizable mitigation strategies are a more likely path to conservation success than general policies. We mentioned that the concept of the population-level UD and its characteristics can help identify measures for mitigating cat impacts through altered overall space-use patterns. However, a conservation measure’s success hinges not only on its effectiveness in achieving the desired results (e.g., supressed spatial coverage or reduced predation on wildlife) if implemented, but depends on the willingness of people—in this case cat owners—to adopt the measure in the first place^37^. Involving cat owners in research on cat ecology and behavior provides an opportunity for behavioral prioritization^38^. This is the process whereby most impactful behaviors are identified, something that should occur before mitigation strategies are decided upon^37^. Here, the catscape can serve another role, namely to relate to cat owners the combined spatial representation of all free ranging pet cats in and around their neighborhood. We suspect that most owners have not thought about and are not aware of the significant population their pet is a part of in a spatial sense. Whether this new perspective and information will prompt owners to recognize the potential ecological implications of their pet as problematic remains uncertain. There are discouraging reports about the ability of factual information on prey return rates to sway attitudes regarding negative environmental impacts of cats among cat owners in the UK^39^.

## Conclusions

We can view animal populations as undulating surfaces draped over the landscape and changing with time^40^. These surfaces represent the combined intensity of use by all individuals in the population, as well as their potential impact on and interactions with the environment. As our study demonstrates, this understanding of populations is useful both from a theoretical and applied perspective. While intensity of use does not necessarily equate risk of predation, use of an area by cats is prerequisite for predation by cats. Studies are needed that can quantify the link between spatially explicit predation risk and the population-level utilization distribution of outdoor pet cats. Our investigation ultimately represents a case study on a single local population of cats. We urge implementation of comparative studies that estimate the population-level utilization distribution for cat populations with different intrinsic characteristics (e.g., demography, maintenance regimes, proportion sterilized) and extrinsic configurations (e.g., position along the urban-nature gradient).

## Methods

### Study area

The study took place in and around Ås, a small (10,725 inhabitants; 4.73 km^2^) university town in southern Norway made up of campus and a commercial center surrounded by residential areas. The area from which cats were recruited encompassed 1.1 km^2^ (Fig. 2, Fig. S1) of residential area (primarily single and multi-family homes and yards). A fenced railroad dissects the study area (Fig. 2), with the two sides connected by pedestrian underpasses. The study area is surrounded by a mixture of forest, and agricultural fields, with a moderately undulating topography.

### Cat recruitment

We used several methods to recruit as many cat owners in our study area as possible: flyers distributed in every residential mailbox in the study area, an advertisement on a local social-media group, and word-of-mouth. This redundancy in outreach was motivated by the goal to recruit as many of the resident cats as possible. We also identified potential cat owners that had not registered during the initial recruitment campaign by inquiring about other households with cats in registered participants’ neighborhoods. These additional households with cats were approached directly with an invitation to participate.

Participants registered for the study through an online registration form, which in addition served as a questionnaire for collecting basic information about the cat(s) (e.g., number of cats with outdoor access) and relevant features of the household (e.g., the owner’s contact information). Once registered, participants completed a follow-up online questionnaire collecting detailed information about each cat and its maintenance.

### GPS tracking

GPS tracking took place between May 1 and May 29, 2021. Owners were instructed to affix the collar with the GPS unit to their cat each time the cat exited the home. We used i-gotU GT-120 GPS units (Mobile Action Technology, Inc., Taiwan) weighing 26 g and requiring manual download. GPS units were set to attempt one position fix every 30 seconds while on. Such high-throughput telemetry data are becoming increasingly common and are particularly useful for revealing detailed movement patterns^41^. Data were stored on-board and downloaded by the authors at least once during the tracking period (to ensure proper functioning, rectify errors in tagging/data collection, backup GPS data) and at the end of it. Participating cat owners were also provided with a hotline for addressing technical problems and to replace lost GPS units.

Owners were instructed to affix the collar with the GPS unit to their cat each time the cat exited the home and to keep the units off and charging while the cat was indoors. This was done to a) ensure batteries were kept charged and b) avoid collecting positions while indoors. However, consistent collaring and collar removal were not always feasible (especially for cats with free access to the outdoors through a pet door). Inspection of the resulting data and conversations with owners indicated that some units were kept powered on while cats were indoors (we attempted to remove these positions, see “Delineation of outdoor activity” below) and that cats occasionally exited without an active collar.

### Camera trapping

We placed one wildlife camera trap with infrared flash in the yards of 47 participating cat owners. The camera models we used were Browning Dark Ops HD Pro Trail Camera BTC-6HDP (37), Browning BTC-6HDPX Dark Ops HD Pro (8) and Browning Spec Ops Full HD (2). Cameras were set 0.5–1 m above the ground and in places that would maximize the probability of recording wildlife and domestic cats entering or moving across the yard, while also protecting the privacy of neighbours. Cameras were programmed to record 10 s videos each time they were triggered. Cameras were placed roughly one week after the GPS tracking started, and remained in the gardens for five weeks. Due to memory limitations, cameras were operational for an average of 22.6 days (sd 12.4). For each cat video, we recorded if the cat wore a GPS collar or not, in order to gauge GPS-coverage of cat activity in the study area during GPS tracking.

### Data analysis

We used R v 4.1.1^17^ for the data processing and the analysis outlined below.

#### GPS data pre-processing

GPS data from each cat were subjected to the following sequence of pre-processing steps, based partially on recommendations by Gupte et al.^42^ and Morris and Conner^43^ :

1. Removed positions with an elevation outside the range 0–300 m.
2. Removed positions obtained during the first 2 days of tracking.
3. Removed positions obtained on days where the GPS was picked up for data download.
4. Removed positions with an estimated horizontal position error (EHPE) $>= 5000$.

#### Delineation of outdoor activity

Although owners were instructed to remove and switch off the GPS collar when the cat was indoors, we know from previous surveys (unpublished data) that this was not done reliably in every household, especially for cats that exited and entered the house freely through a cat flap or similar. GPS data revealed a high degree of clustering, particularly in the vicinity of the cat’s home. We used R package GPSeqClus^44^ to identify sequential position clusters based on a reported gps error <10 m^43^. As we were interested in outdoor activity, we identified GPS clusters with centroids that fell within the spatial extent of the owner’s residence (building spatial data obtained from the Norwegian Mapping Authority) and then removed all relocations associated with these clusters. This ensures that positions used during subsequent analyses can be considered arising from a cat’s outdoor activity.

#### Individual utilization distributions

We used R package BBMM^45^ to construct a Brownian bridge movement model^46,47^ for each cat in the study. We chose BBMM, as it is better equipped to deal with auto-correlated and clustered position data than the kernel density home range estimator commonly used in wildlife studies^48^. Specifically, BBMM allows estimation of probabilistic space-use, while accounting for position error and the uncertainty about paths taken between consecutive positions^47^. Prior to constructing BBMMs, we thinned GPS data to yield position fix interval $>= 2$ min.

#### Population-level utilization distribution

With some modification, we used the approach outlined by^47^, and subsequently followed by^49^ and^50^, to construct the population-level utilization distribution (UD) from individual UDs (BBMM). This involved the following steps (see also Fig. 1):

1. Resample individual UD rasters to align (same spatial extent and resolution).
2. Re-scale cell values in individual UD rasters to sum to the average proportion of a day the cat was tracked outdoors on days with outdoor position data. This assumes that (a) cats were tracked for the entire time they spent outdoors and (b) on days without any GPS tracking data, cats spent an average amount of time outdoors, but were not tracked. We know from simultaneous camera trapping data that the former assumption was violated, whereas the latter assumption likely held, based on communications with owners. In general, this means that outdoor utilization was underestimated and that the final population-level catscape represents a minimum use scenario.
3. Aggregate individual UD rasters into a population-level catscape by summing cell values across the stack of 92 weighted individual UD rasters^47^. Unlike^47^, we did not perform further scaling of the resulting raster to sum to 1. This allows comparison of population-level UDs within (e.g., day vs. night) and between populations in terms of both their magnitude and shape.

### Ethics declarations

We used data generated by commercially available GPS-trackers intended for cats and similar-sized pets that were voluntarily deployed by individual cat owners as parts of their private cat maintenance regimes. No cats were handled by the research team and pet-tracking was outside the purview of the Norwegian University of Life Sciences animal use committee and the Norwegian government agency (Norwegian Food Safety Authority; Mattilsynet) responsible for approval of protocols involving experiments with animals. All methods were carried out in accordance with relevant guidelines and regulations at the Norwegian University of Life Sciences and according to Norwegian law.

## Supplementary Information

Supplementary Information.

## Data Availability

The cat GPS data used in this manuscript are available on GitHub (https://github.com/richbi/CatTrack_public). Note that a random offset has been added to positions in order to protect the privacy of the owners of the cats.
