# Supplementary material for: Mapping the “catscape” formed by a population of pet cats with outdoor access
Source: Sci Rep. 2022 Apr 8;12:5964. doi: 10.1038/s41598-022-09694-9 (PMC8993881; doi:10.1038/s41598-022-09694-9)
Supplement: Supplementary file 1 — Supplementary Information. [file 41598_2022_9694_MOESM1_ESM.pdf]

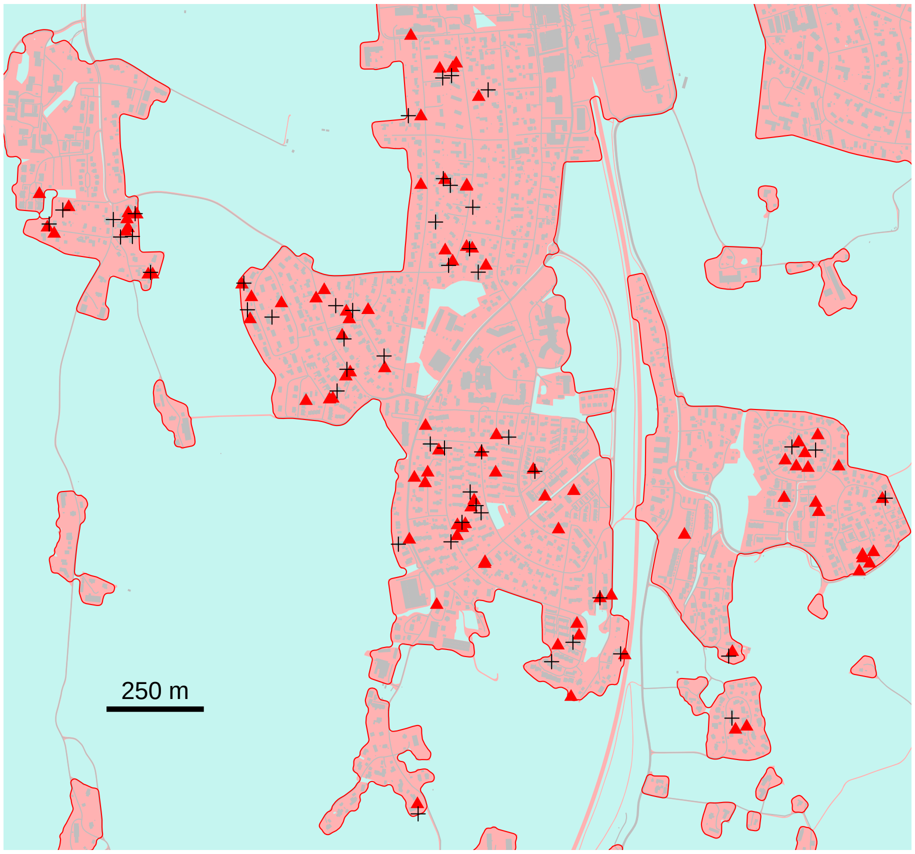

Figure S1. Map of the study area showing the distribution of 47 camera traps (black crosses) used to monitor domestic cats. Camera trap locations are shown in the context of developed areas, including roads (pink), pastures, fields, and forests (turquoise). The red line marks the urban boundary (including scattered buildings/farm yards on the periphery) used for distance calculations in the analysis. Buildings are shown in grey, with the approximate location of the homes of 92 GPS-tagged cats indicated with red triangles. The locations of camera traps and cat homes are shown with a random error (50 m radius) to protect the privacy of study participants. Camera trapping resulted in 2797 video captures of cats, with a mean of 65 captures per camera (range: 1–301 captures). The map was created in R (R Core Team. R: A Language and Environment for Statistical Computing R Foundation for Statistical Computing[1] (Vienna, Austria, 2021).
